# Fourteen simple-sequence repeats newly developed for population genetic studies in *Prosopis africana* (*Fabaceae*–*Mimosoideae*)

**DOI:** 10.1186/s13104-017-2755-x

**Published:** 2017-08-31

**Authors:** Guibien Cleophas Zerbo, Heino Konrad, Moussa Ouedraogo, Thomas Geburek

**Affiliations:** 1 Department of Forest Genetics, Austrian Research Centre for Forests, Natural Hazards and Landscape, Seckendorff-Gudent-Weg 8, 1131 Vienna, Austria; 2 Centre National de Semences Forestières, Ouagadougou, 01 BP 2682 Ouagadougou, Burkina Faso

**Keywords:** African mesquite, Genetic characterization, Microsatellites

## Abstract

**Background:**

There is very limited genetic knowledge in *Prosopis africana,* an important sub-Saharan multi-purpose tree species. Availability of highly polymorphic genetic markers would be helpful for future genetic work.

**Findings:**

Leaf samples from 15 trees were used to develop simple sequence repeat (SSR) markers. Size-selected fragments from genomic DNA were enriched for repeats and the library was analyzed on an Illumina MiSeq platform. Fourteen SSRs were selected and applied in two Burkinabe populations (40 adult trees each). The number of alleles varied from 4 to 20, evenness (effective number of alleles/observed number of alleles) averaged to 0.54 and unbiased heterozygosity ranged from 0.305 to 0.925 over all loci and populations. Null alleles were not detected.

**Conclusions:**

Due to the high level of polymorphism and lack of null alleles the developed SSRs can be effectively employed in population genetic studies.

**Electronic supplementary material:**

The online version of this article (doi:10.1186/s13104-017-2755-x) contains supplementary material, which is available to authorized users.

## Introduction

African mesquite (*Prosopis africana* [*Fabaceae*: *Mimosoideae*]) is a valuable, medium-sized (up to 20 m height), multi-purpose tree in sub-Saharan Africa. It is the only species of the genus *Prosopis* native to Africa and occurs at sites with 600–1500 mm annual rainfall [1] (and references therein). The modeled species distribution covers savannas and dry forests of tropical Africa within an approximately 500 km wide band from Senegal to Sudan (Gaisberger et al. unpublished results). The hard wood has a high calorific value making it highly valuable as fuel wood and for charcoal production. The leaves, bark and roots are used for various medicinal purposes. Its pods are preferred fodder for livestock and wildlife. Seeds are dispersed endozoochorously and germinate freely after passing through the digestive system of ungulates. Fermented seeds serve as seasoning ([1] and references therein).

Genetic knowledge of African mesquite is very scarce as only results from a single provenance trial with material originating from Niger and Burkina Faso are at hand. Survival, growth and wood density seem to be related to humidity of the seed source [1, 2]. So far no specific genetic markers have been available for *P. africana*. However, simple-sequence repeat markers (SSRs) were developed for other *Prosopis* species [3–5]. Unfortunately, cross-species amplification of SSRs developed by Mottura et al. [4] was not successful (Zerbo et al. unpublished results). Hence, the major objective of this study was to develop highly polymorphic SSRs for this species.

## Materials and methods

### Materials

For primer development DNA was extracted from leaves collected from adult trees in two populations from Burkina Faso. Twelve individuals were selected as screening panel in Yeimzuro (13°36′40.07″N, 2°9′44.30″W) and three in Padiali (11°8′35.50″N, 0°48′55.60″E). An emphasis was put on selecting more trees in Yeimzuro due to its location in the North of Burkina Faso as less diversity is expected in that region as observed by Schmidt et al. at the species level [6].

The polymorphism of the developed markers was tested and the population structure was estimated using leaves collected from 40 trees per site in two other populations: Raguitenga (12°47′2.74″N, 1°6′55.88″W) and Bandougou (10°58′43.90″N, 4°51′24.43″W). The former is located in the Sudano-Sahelian climatic zone with a savannah type of vegetation and tree density is low; in the latter, tree density is high and the site is located in the Sudanian climatic zone with dry forest vegetation type. These populations are separated by about 500 km, occurring in two different climatic zones, which were chosen to obtain a better genetic diversity estimate of the species over a larger area.

### DNA extraction and SSRs development

DNA was extracted using the DNeasy Plant Mini Kit and the DNeasy 96 Plant Kit (QIAGEN, Hombrechtikon, Switzerland) following the manufacturer’s protocols. SSRs were developed by Ecogenics (Balgach, Switzerland). Size-selected fragments from genomic DNA were enriched for SSR content by using magnetic streptavidin beads and biotin-labeled CT and GT repeat oligonucleotides. The SSR-enriched library was analysed on an Illumina MiSeq platform using the Nano 2 × 250 v2 format. After assembly, 3′635 contigs or singlets contained a microsatellite insert with a tetra- or a trinucleotide of at least 6 repeat units or a dinucleotide of at least 10 repeat units. Suitable primer design was possible in 2′232 microsatellite candidates by Ecogenics (Balgach, Switzerland) using the Primer3 software [7]; for subsequent analysis 14 random loci were selected which was deemed a sufficient number for population genetic studies. To determine polymorphisms of these newly developed markers, the approach originally described by Schuelke [8] was used by adding a universal 18 base pair M13 tail to the 5′-end of forward primers. Multiplex PCR amplification was optimized to be performed in a 10 μl reaction volume containing 2–10 ng of genomic DNA, 5 μl HotStarTaq Master Mix (Qiagen), double distilled water, and 0.1–0.3 µM of forward and reverse primer each. The following cycling protocol on a TC-412 programmable thermal controller (Techne) was used: 35 cycles with 94 °C for 30 s, 56 °C for 90 s, and 72 °C for 60 s. Before the first cycle, a prolonged denaturation step (95 °C for 15 min) was included and the last cycle was followed by a 30 min extension at 72 °C. For determination of allele sizes on an ABI3730 (applied biosystems) M13 primers were labelled either with Atto565, Atto550, Atto532 (Sigma Aldrich), or FAM (applied biosystems) and an internal size standard (LIZ500; applied biosystems) was added.

### Statistical analysis of genetic parameters

Standard genetic parameters were estimated with GenAlEx 6.5 [9]. Micro-Checker version 2.2.3 [10] was used to test the presence of null alleles. Linkage disequilibrium (LD) was analysed by Genepop V4.4 [11, 12] setting Markov chain parameter to 10 000 for the dememorization number, 100 for the number of batches with 5000 iterations per batch. To detect possible population size reduction, the program BOTTLENECK V1.2.02 was used [13]. The infinite alleles model (IAM), the stepwise mutation model (SMM) and the two-phase mutation model (TPM) were applied using 70% of SMM in TPM with 1 000 iterations to perform the Wilcoxon test which produce the most reliable results [14].

## Results and discussion

### SSRs characterization

The 14 newly developed primers were utilized for further analysis of two Burkinabe populations (Raguitenga and Bandougou). All the fourteen tested SSRs were polymorphic for 2-bp perfect tandem repeats and the number of alleles ranged from 6 to 14 in the screening panel. The characteristics of the developed markers are summarized in Table 1.

**Table 1:** Description of 14 SSRs developed for *Prosopis africana*

Locus	Primer sequences 5′–3′		Repeat type^a^	Size bp^b^	No of alleles^b^	Genbank accession number
Proafr_01109c	F	TGATCGCGTTGTTTACTTTTGC	(TG)_14_	162–185	6	KU726842
	R	GGGCTTGGTCCAGTTGTTATC				
Proafr_01293c	F	TTTGGAGCAGTAACGCAAGC	(AG)_16_	149–194	12	KU726843
	R	AAAAGGCTAAACGGACTGGG				
Proafr_01664c	F	TGGACAAATCAAGCCTTATCACC	(AC)_13_	133–157	7	KU726844
	R	AGCATGTTCGTATTGTTGCC				
Proafr_03430c	F	ACAGTTTCCGGTGATACTCATTC	(AG)_15_	232–253	10	KU726845
	R	TGGCCAATACAACGGGAAG				
Proafr_03572c	F	AAAGCGTCATGAGACCAAGC	(TC)_17_	232–253	5	KU726846
	R	GAAGCTGTTTGGTCAGCCAC				
Proafr_05728c	F	GTCCACATTTCTGAAGACACCC	(CT)_13_	124–140	7	KU726847
	R	ACACGTGGTTTAATCTGATGC				
Proafr_08290c	F	GGCTCAAGCCCTGAAACATAC	(CT)_16_	168–210	11	KU726848
	R	ATTTGGAAAGAGCCACCTCC				
Proafr_08425c	F	TTTCCTACGACGCTCCCATC	(CT)_14_	203–221	8	KU726849
	R	ACGATGCTAACGTCTCTTTTGG				
Proafr_09196c	F	TGTCTTTCGAACCCTATTAGCAC	(TC)_15_	251–266	6	KU726851
	R	ACTTGACAAAGGAAAATTAAGGCG				
Proafr_10663c	F	CACCTCTATAATATGTGCGTGC	(AG)_14_	081–114	8	KU726852
	R	ACTTTCACTAAGTTGCCCCTAC				
Proafr_11069c	F	TTTGTTCAGCGTAGCCTGTC	(AC)_13_	218–234	6	KU726853
	R	GACCGACAAATGAAGTCCCAG				
Proafr_11635s	F	TTGGCGCAAAAATGGAGGTC	(AG)_16_	132–144	6	KU726854
	R	ATGCATCGTCCTATTCCCCC				
Proafr_12199s	F	GCGTTTGACAACTGCGTAGC	(TC)_16_	129–173	14	KU726855
	R	TGCAACTGGGGAAGATTTATGTG				
Proafr_12745s	F	TTAGGCAAGAGATCCCCGTG	(CT)_14_	205–225	8	KU726856
	R	AGCTTGTGGTCGTGGATTTG				

^a^Based on genomic DNA sequence analysed on a Illumina MiSec platform

^b^Based on the fragment analysis of 15 individuals on an ABI3730

### Genetic characterization of populations

In both populations investigated to test the usefulness of these markers for genetic analysis (Raguitenga and Bandougou) all 14 loci were polymorphic (Table 2). The number of alleles ranged from four to 21. Neither null alleles nor linkage disequilibrium were detected between locus pairs after Bonferroni corrections. The average fixation index over all populations was close to zero.

**Table 2:** Population genetic parameters based on 14 SSRs developed for *Prosopis africana*

Locus	Raguitenga								
	N	N_a_	Mnsr	N_e_	N_e_/N_a_	H_o_	uH_e_	HWE	F
Proafr_01109c	40	8	22	4.48	0.56	0.800	0.786	ns	−0.030
Proafr_10663c	40	9	22	6.13	0.68	0.925	0.847	ns	−0.105
Proafr_11069c	38	4	20	1.43	0.36	0.237	0.305	ns	0.213
Proafr_01664c	40	7	21	5.17	0.74	0.850	0.817	ns	−0.054
Proafr_08290c	40	17	35	8.08	0.48	0.850	0.887	ns	0.030
Proafr_05728c	40	8	21	3.07	0.38	0.675	0.683	ns	−0.001
Proafr_08425c	34	10	24	4.83	0.48	0.765	0.805	ns	0.035
Proafr_03430c	28	11	25	4.45	0.40	0.607	0.790	**	0.217
Proafr_12199s	39	20	45	11.57	0.58	0.923	0.925	ns	−0.010
Proafr_11635s	40	6	22	4.44	0.74	0.875	0.785	ns	−0.129
Proafr_09196c	40	9	26	3.24	0.36	0.775	0.700	ns	−0.121
Proafr_12745s	40	10	22	4.79	0.48	0.775	0.801	ns	0.021
Proafr_03572c	40	7	22	3.96	0.57	0.800	0.757	ns	−0.071
Proafr_01293c	40	12	34	6.15	0.51	0.925	0.848	ns	−0.104

Locus	Bandougou									Total N_a_
	N	N_a_	Mnsr	N_e_	N_e_/N_a_	H_o_	uH_e_	HWE	F	
Proafr_01109c	40	7	22	3.98	0.57	0.675	0.758	ns	0.098	9
Proafr_10663c	40	7	22	5.37	0.77	0.875	0.824	ns	−0.075	9
Proafr_11069c	34	4	20	2.69	0.67	0.735	0.637	ns	−0.172	4
Proafr_01664c	40	6	20	4.15	0.69	0.800	0.769	ns	−0.054	8
Proafr_08290c	40	12	35	4.55	0.38	0.725	0.790	**	0.071	19
Proafr_05728c	40	10	23	5.25	0.53	0.750	0.820	ns	0.074	11
Proafr_08425c	39	8	24	2.68	0.34	0.513	0.636	ns	0.183	10
Proafr_03430c	40	9	23	5.77	0.64	0.850	0.837	ns	−0.028	12
Proafr_12199s	40	16	33	9.47	0.59	0.900	0.906	ns	−0.006	21
Proafr_11635s	40	7	23	4.31	0.62	0.775	0.778	ns	−0.009	7
Proafr_09196c	40	8	26	2.20	0.28	0.500	0.553	***	0.084	9
Proafr_12745s	40	12	24	5.57	0.46	0.825	0.831	ns	−0.005	13
Proafr_03572c	40	8	23	4.25	0.53	0.700	0.774	***	0.085	9
Proafr_01293c	40	12	34	6.05	0.50	0.825	0.845	***	0.012	17

*N* number of individuals genotyped, *N*_*a*_ number of alleles, *Mnsr* maximum number of sequence repeats, *N*_*e*_ effective number of alleles, *H*_*o*_ observed heterozygosity, *uH*_*e*_ unbiased expected heterozygosity, *HWE* Hardy–Weinberg expectation, *ns* not significant, *F* fixation index

* P < 0.05, ** P < 0.01, *** P < 0.001

**Fig. 1:**
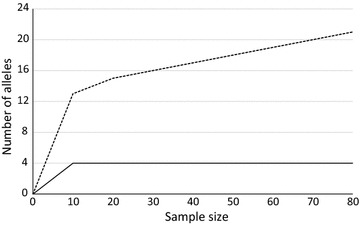
Number of alleles detected in relation to the sample size for the most (Proafr_12199s *dotted line*) and the least polymorphic locus (Proafr_11069c *solid line*)

When the sample size was progressively increased from 10 to 80 (all individuals studied), the number of alleles remained quite constant at four in the locus with the lowest number of alleles (Proafr_11069c), while this number ranged from 10 to 21 for Proafr_12199s, which was the locus with the largest number of alleles (Fig. 1). It was thus concluded that sample sizes of 50 individuals are sufficient for population genetic analysis. For a paternity analysis utilizing the developed markers it is particularly useful to know how many loci will be required. Using only the first four loci from Table 2 (showing a moderate number of alleles) for paternity analysis, the exclusion probability for excluding a putative parent pair already amounted to 0.998; therefore already a subset of the markers will be adequate for paternity analysis. Using all 14 loci the exclusion probability in both populations studied was larger than 0.9999.

The Mnsr (maximum number of sequence repeats) per locus ranged from 20 to 45 in Raguitenga and from 20 to 35 in Bandougou indicating the potential finding of additional alleles in other *P. africana* populations. In total five loci (one in Raguitenga and four in Bandougou) showed significant deviation from HWE (Hardy–Weinberg expectation). While the Raguitenga population consisted of even-aged mature trees, in Bandougou different age classes were sampled; therefore we expected a higher number of deviations in Bandougou as in tree species with mixed-mating young cohorts often deviate more strongly from HWE (e.g., [15]).

The evenness of the allele distribution (N_e_/N_a_) which theoretically ranges from 0 (lack of evenness) to 1 (complete evenness) varied from 0.28 (Proafr_09196c) to 0.77 (Proafr_10663c) with an average value of 0.5 for each population. At least seven loci showed an evenness value above the average evenness. Loci with a high evenness and high number of alleles should be selected for the analysis when the number of loci is restricted [16].

Generally the degree of polymorphism detected in our data was high. The number of alleles and unbiased heterozygosity was much higher in our populations than in those developed for *P. alba*, *P. chilensis*, *P. flexuosa*, *P. rubriflora* and *P. ruscifolia* [3–5]. However, we should keep in mind that the sample sizes were smaller in these studies (<20 individuals per population).

Both populations showed bottleneck effects (P < 0.05) under the IAM and only Raguitenga under the SMM (Table 3). According to Cornuet and Luikart [17] the SMM is the most conservative model for testing significant heterozygosity excess caused by a bottleneck. Raguitenga is located in a dry area where generally few tree species are found at a low density. *Prosopis africana* is overexploited in this area leading to a reduction of its population size. Therefore the observed bottleneck effect in this population was not unexpected.

**Table 3:** Wilcoxon test results for the three models IAM, TPM and SMM

Population	P__IAM_	P__TPM_	P__SMM_
Bandougou	0.020^*^	0.502	0.058
Raguitenga	0.013^*^	0.761	0.017^*^

* P < 0.05

## Conclusion

All our 14 newly developed markers were highly polymorphic and no null alleles were detected. Using these SRRs, it was possible to quantify the genetic structure of two populations. These SSRs are very valuable for population genetic studies including the analysis of the mating system and gene flow parameters especially when markers which will be employed having a high N_a_ and a high N_a_/N_e_-ratio.

## Additional file

**Additional file 1: Appendix 1.** Information on samples of *Prosopis africana* used in the study. Table giving information on the samples used in the study.
