# Supplementary material for: Fourteen simple-sequence repeats newly developed for population genetic studies in Prosopis africana (Fabaceae–Mimosoideae)
Source: BMC Res Notes. 2017 Aug 31;10:437. doi: 10.1186/s13104-017-2755-x (PMC5580295; doi:10.1186/s13104-017-2755-x)
Supplement: Supplementary file 1 — Additional file 1: Appendix 1. Information on samples of Prosopis africana used in the study. Table giving information on the samples used in the study. [file 13104_2017_2755_MOESM1_ESM.docx]

Appendix 1: Information on samples of *Prosopis africana* used in this study.

| Species | Accession no. and type^a^ | Collection locality ^b^ | Geographic coordinates | No. of individuals in the herbarium | No. of individual used for testing primers for polymorphism | No. of individuals used for characterization of developed markers |
| --- | --- | --- | --- | --- | --- | --- |
| *P. africana* | Pa-ZG001  Vouchers | Yeimzuro, Titao, Loroum | 13°36’40.07’’N,  2°9’44.30’’W | 12 | 12 | 0 |
| *P. africana* | Pa-ZG002  vouchers | Padiali, Pama, Kompienga | 11°8’35.50’’N,  0°48’55.60’’E | 3 | 3 | 0 |
| *P. africana* | Pa-ZG003  dried leaves | Raguitenga, Korsimoro, Sanmatenga | 12°47’2.74’’N,  1°6’55.88’’W | 40 | 0 | 40 |
| *P. africana* | Pa-ZG004  dried leaves | Bandougou, Orodara, Kenedougou | 10°58’43.90’’N,  4°51’24.43’’W | 40 | 0 | 40 |

^a^ Plant material was collected by Guibien Cleophas Zerbo. Both vouchers and dried leaves are stored at the herbarium at the Centre National de Semences Forestrières, Route de Kaya, Ouagadougou, Burkina Faso.

^b^ Village, department and province in Burkina Faso
